# Efficacy of fungi-degraded date pits as a potential alternative dietary energy source in farm animal diets

**DOI:** 10.3389/fvets.2026.1892086

**Published:** 2026-10-21

**Authors:** Sobhy M. A. Sallam, Marwa F. A. Attia, Amr E. El-Nile, Mahmoud El-Azab, Mohamed A. Shehab El-Deen, Mohamed Aladhadh, Ahmed Z. Ghareeb, Elsyed E. Hafez, Ahmed M.M. Salama, Doaa A. Ghareeb

**Affiliations:** 1 Animal and Poultry Production Department, Faculty of Agriculture and Food, Qassim University, Buraydah, Al-Qassim, Saudi Arabia; 2 Agricultural Research Center, Animal Production Research Institute, Giza, Egypt; 3 Livestock Research Department, Arid Land Cultivation Research Institute, City of Scientific Research and Technological Applications, New Borg El-Arab, Alexandria, Egypt; 4 Centre of Excellence for Drug Preclinical Studies (CE-DPS), Pharmaceutical and Fermentation Industry Development Centre, City of Scientific Research and Technological Applications, New Borg El-Arab, Alexandria, Egypt; 5 Department of Plant Protection and Bimolecular Diagnosis, Arid Lands Cultivation Research Institute, City of Scientific Research and Technological Applications, New Borg El-Arab, Alexandria, Egypt; 6 Botany and Microbiology Department, Faculty of Science, Alexandria University, Alexandria, Egypt; 7 Biochemistry Department, Faculty of Science, Alexandria University, Alexandria, Egypt

**Keywords:** agricultural byproducts, biological treatment, feed shortage, global warming, rumen fermentation

## Abstract

Date pits (DPs) are a rich and economical source of dietary energy, and they show promise as an alternative to expensive concentrates in cattle feeding. This study examined the effect of replacing yellow corn grain with different levels of date pits treated with *Aspergillus parasiticus* on *in vitro* rumen fermentation characteristics, nutrient degradability, methanogenic community, and methane production using a semi-automatic system of gas production (GP). In the *in vitro* experiment, a basal diet (50% concentrate mixture and 50% clover hay) was incubated without date pits (control) and with replacement ratios (25, 50, 75, and 100%) of fungi-degraded date pits (FDDPs). Linear and quadratic reductions (*p* = 0.001) in gas production (GP), truly degraded dry matter (TDDM), and truly degraded organic matter (TDOM) values were observed (*p* = 0.001), with increasing levels of FDDP supplementation. However, FDDP supplementation did not affect CH_4_, PF, total protozoa, NH_3,_ or the molar proportions of the main short-chain fatty acids (SCFAs). The majority of the identified methanogen sequences grouped within the *Methanobrevibacter* lineage, according to phylogenetic analysis. Bootstrap values ranging from 39 to 67% confirmed the observed clustering patterns and demonstrated the evolutionary relatedness of these sequences; there were no notable differences in methane production when using varying levels of FDDP. It might be concluded from the results of this study that fermented date pits are a promising alternative to corn grain, with no detrimental effects on rumen fermentation characteristics and the methanogenic community in the feeding of ruminants.

## 1 Introduction

Recently, the cost of energy supplements used in animal feeds has increased dramatically because of growing demand, which drives nutritionists to search for cheaper alternative high-energy feed ingredients (1). Additionally, corn and barley are well known to be widely used in animal feeds due to their high availability and digestibility. This has driven growing interest in local agro-industrial by-products as alternative feed ingredients, particularly those that do not compete with human food consumption, since they can help address feed shortages while minimizing environmental impact (2). This approach is especially feasible for ruminants, which are able to utilize high-fiber diets effectively (3). Date palms are primarily cultivated in the hot regions of the Middle East and Africa. Egypt is considered the world’s largest producer of dates, accounting for nearly 21% of global production or approximately 1.7 million metric tons, according to FAO data from 2020 (4).

Date pits (DPs), which account for approximately 13–15% of the total weight of dates, are produced in large quantities during date processing. In Egypt alone, this corresponds to approximately 189,000 metric tons annually (1). Without proper utilization or treatment, this by-product may cause environmental issues, highlighting the need for value-added applications. Date pits (DPs) are rich in carbohydrates and represent an important energy source. They also contain structural fiber components, such as cellulose, hemicellulose, and lignin, which are relevant for ruminant diets (5). Alyileili et al. (6) stated that degraded DPs contain moderate levels of crude fiber (20.8%), crude fat (7.2%), and crude protein (5.56%). They also contain a total carbohydrate content of 87.2%, along with relatively high neutral detergent fiber (NDF; 74.6%) and acid detergent fiber (ADF; 45.7%) values, indicating their fibrous nature.

Therefore, DPs can serve as a good alternative feed ingredient in concentrate formulations without compromising the productive efficiency of ruminants. The incorporation of date pits into a diet based on wheat straw and concentrate has a positive effect on animal weight improvement and, consequently, on average daily weight gain. It should be noted that date seeds constitute a good source of cellulose and, consequently, energy (7). Sami et al. (8) reported that substituting up to 50% of barley with crushed date pits, a low-quality and inexpensive energy feedstuff, supplemented with a commercial enzyme mixture as a natural growth promoter, in the diets of Naimi growing lambs resulted in acceptable carcass characteristics and desirable meat quality attributes. Rabee et al. (2) demonstrated that incorporating olive cake and date palm by-products into animal diets influenced animal performance, rumen fermentation, microbial diversity, and rumen bacterial populations. In another study, Bakr et al. (7) showed that replacing 10 to 20% of yellow corn and wheat bran with a mixture of date pits and soybean meal had no negative effects on the growth performance, digestibility, blood metabolites, or rumen characteristics of Barki lambs.

Moreover, Faishal et al. (9) reported that date seed flour can be incorporated into concentrate mixtures for dairy cows at levels up to 15% without negatively affecting dry matter (DM) digestibility or blood profile, while also significantly improving DM digestibility. Although date seed flour can be incorporated into concentrate diets at levels up to 15%, it does not negatively affect dairy cows’ digestibility and blood profile. Overall, many studies have explored the use of date pits in livestock feeding (43, 45). However, information regarding their effects on the rumen microbiota remains limited (10, 44).

Bacteria are the dominant microorganisms in the rumen and play a major role in rumen fermentation (11). They ferment various dietary components such as cellulose, xylan, amylose, and proteins, producing short-chain fatty acids (SCFAs) and microbial protein, which provide animals with a substantial proportion of their energy and protein requirements (12). The composition of rumen bacteria is greatly influenced by diet, since dietary composition affects rumen fermentation and pH (13). High-fiber diets stimulate fibrolytic bacteria, while diets rich in starch and soluble sugars promote amylolytic bacteria (14). Therefore, gaining a better understanding of the rumen microbiome could help improve animal productivity (12).

High-throughput sequencing technologies have significantly improved our understanding of rumen microbial communities. These techniques have also enabled researchers to study how microbial populations vary in response to changes in diet and geographic location (14). However, the accuracy of the results may still be affected by factors such as DNA extraction methods, PCR primers, sequencing platforms, and bioinformatics analysis pipelines (12). Therefore, it is highly recommended to link rumen microbiota, functional genes, metabolic pathways, rumen metabolites, and animal performance (15). Nevertheless, to the best of our knowledge, information regarding the effects of fungi-degraded date pits (FDDPs) on the methane-producing community in the rumen is extremely limited. We hypothesized that FDDP supplementation could be an economical, high-quality feed energy replacement source. Therefore, the objective of this study was to assess the use of a biotechnological method involving date pits (an agricultural waste material) treated with *Aspergillus parasiticus* (FDDP) and its effects on *in vitro* rumen fermentation characteristics, nutrient degradability, and the methanogenic community.

## 2 Materials and methods

The study was carried out at the Advanced Laboratory of Animal Nutrition and the Experimental Farm, Faculty of Agriculture, Alexandria University, and the Laboratory of Preclinical Research Studies, Department of Pharmaceutical and Fermentation Industries Development Center, City for Scientific Research and Technological Applications, Alexandria, Egypt. All procedures were approved and authorized by the Institutional Animal Care and Use Committee of Alexandria University (protocol ID: Alex. Agri. 082408446).

### 2.1 Preparation of fungi-degraded date pits (FDDP)

Freshly separated date pits were cleaned thoroughly with tap water, followed by rinsing with distilled water, to remove adhering impurities. The cleaned date pits were then dried in an oven at 80 °C for 8 h. After drying, the date pits were finely ground using a feed grinder and passed through a 0.6 mm diameter sieve to obtain degraded date pit (DDP) powder. *Aspergillus parasiticus* was cultured on potato dextrose agar (PDA) plates. The PDA medium consisted of potato infusion (200 g), dextrose (20 g), and agar (20 g) dissolved in 1 L of distilled water, with a final pH of 5.6 ± 0.2. The fungal cultures were incubated at 30 °C for 7 days in the dark. The FDDP was produced using a solid-state fermentation (SSF) process in which simple autoclavable bags were used. At first, the autoclavable bags, substrate (degraded date pits; DDPs), and the medium were cleaned and sterilized by autoclaving at 121 °C for 20 min on three consecutive days.

Approximately 25 kg of ground date pits were then inoculated with 40 agar plugs (6 mm diameter) obtained from the actively growing margins of *Aspergillus parasiticus* colonies, followed by the addition of 50 L of the medium. The medium was adjusted to pH 5.5, with agitation at 200 r.p.m., at 37 °C, and supplemented with 2.5% lactose. In addition, 1 L of the medium contained the following: malt extract 1%, 1.89 mM (NH₄)₂SO₄, 10.29 mM KH₂PO₄, 18.02 mM CaCl₂, 1.21 mM MgSO₄·7H₂O, 35.29 mM NaNO₃, 6.70 mM KCl, and trace elements including 2.01 μM CuSO₄·5H₂O, 1.79 μM FeSO₄·7H₂O, 7.17 μM MnSO₄·4H₂O, 4.86 μM ZnSO₄·7H₂O, and 84.05 μM CoCl₂·6H₂O, along with 0.1% Tween 80 (v/v). (pH 5.5), agitation 200r.p.m., at 37 °C and 2.5%lactose). The autoclavable bags were incubated at 28 °C in the dark for 2 weeks. During the degradation process, continuous aeration was needed because the process is primarily aerobic. Cooling was also necessary because large amounts of heat evolved during microbial growth. Stirring was needed to intensify cooling and to facilitate air transport to the organisms. After incubation, colonized ground date pits or FDDPs had been autoclaved twice.

### 2.2 Aflatoxin determination

Total aflatoxins (AFB1, AFB2, AFG1, and AFG2) were determined in the fermented date pit samples using a commercial enzyme-linked immunosorbent assay (ELISA) kit according to the manufacturer’s instructions (Neogen (Veratox^®^ for Total Aflatoxin), USA). Briefly, 5 g of the dried sample was extracted with 25 mL of methanol–water solution (70:30, v/v) by shaking for 30 min, followed by filtration. The filtrate was diluted as required and analyzed using an ELISA microplate reader at 450 nm. Aflatoxin concentrations were calculated from a calibration curve prepared using aflatoxin standards and expressed as μg/kg (ppb) (16). The aflatoxin level measured was 0.72 ppm, which was below the maximum limits established for animal feed, indicating no apparent safety concerns.

### 2.3 Biosafety measures

Upon completion of the fermentation process, the cultivation bags were exposed to ultraviolet (UV) radiation for 1 h prior to opening. This procedure was implemented to reduce viable fungal spores and minimize potential environmental dissemination during sample handling.

### 2.4 Experimental diets and treatments

In total, five isocaloric and isonitrogenous total mixed rations (TMRs) were formulated to meet the nutrient requirements of lactating dairy cows according to the NRC (17) guidelines. Each diet consisted of 50% concentrate mixture and 50% clover (*Trifolium alexandrinum*) hay and served as the incubated substrate for the gas production (GP) assay. Yellow corn was incrementally replaced with FDDPs across five dietary treatments as follows:

1 Control (CTRL): 0% FDDP + 100% yellow corn
2 FDDP_25%_: 25% FDDP + 75% yellow corn
3 FDDP_50%_: 50% FDDP + 50% yellow corn
4 FDDP_75%_: 75% FDDP + 25% yellow corn
5 FDDP_100%_: 100% FDDP + 0% yellow corn

The ingredients and chemical composition of FDDPs and experimental diets on a dry matter basis are shown in Table 1.

**Table 1:** Ingredients and chemical composition of FDDPs and experimental diets on a DM basis, g/kg DM.

		Experimental diets				
Items	FDDP	CTRL	FDDP_25%_	FDDP_50%_	FDDP_75%_	FDDP_100%_
Clover hay	––	500	500	500	500	500
Yellow corn	––	250	187.5	125	62.5	0
Wheat bran	––	82	82	82	82	82
Soybean meal	––	140	140	140	140	140
FDDPs	––	0	62.5	125	187.5	250
Limestone	––	15	15	15	15	15
Sodium chloride	––	10	10	10	10	10
Trace minerals and vitamins*	––	3	3	3	3	3
Chemical composition, g/kg DM						
Dry matter	928.3	906.2	908.4	910.1	913.2	911.9
Organic matter	913.5	873.2	876.2	867.5	867.9	860.4
Ash	86.5	126.8	123.8	132.5	132.1	139.6
Crude protein	85.6	169.8	168.9	167.3	171.3	163.9
Ether extract	34.5	22.2	21.3	18.2	19.8	16.2
NDF	481.3	372	421.1	420.3	461.8	448.2
ADF	331.2	196.5	234.4	245.1	272.4	268.3
ADL	55.58	45.1	49.7	54.9	55.2	59.9
Hemicellulose	150.1	175.5	186.7	175.2	189.4	179.9
Cellulose	275.62	151.4	184.7	190.2	217.2	208.4
NFC	312.1	309.2	264.9	261.7	215	232.1
GE, MJ/kg DM	17.82	15.78	15.87	15.92	16.18	15.97

FDDPs, Fungi-degraded date pits; CTRL, Control diet; NDF, Neutral detergent fibre; ADF, Acid detergent fibre; ADL, Acid detergent lignin; GE, Gross energy; and NFC, Non-fibre carbohydrate, calculated as 1,000- NDF+CP+EE+ Ash.

*Each kg contained 45.8 g dicalcium phosphate, 15 g magnesium sulfate, 6.15 g ferrous sulfate, 0.393 g potassium iodide, 0.753 g copper sulfate, 0.248 g cobalt sulfate, 0.373 g zinc sulfate, and 0.02 g sodium selenite.

### 2.5 *In vitro* gas production procedure

The *in vitro* gas production assay was carried out at the Advanced Laboratory of Animal Nutrition and the Agricultural Experimental Station (31°20′N, 30°E), Faculty of Agriculture, Alexandria University, Egypt. All procedures and experimental protocols were carried out in accordance with Directive 2010/63/EU of the European Parliament and of the Council of 22 September 2010 on the protection of animals used for scientific purposes.

An *in vitro* trial was conducted to evaluate the effects of partial/or total substitution of yellow corn with FDDPs on rumen fermentation, nutrient degradability, fermentation parameters, and total protozoal count using a semi-automatic system of gas production (GP) and molecular characterization of rumen populations.

The *in vitro* GP assay was carried out according to the method described by Theodorou et al. (18), with adaptations for the semi-automatic system of Mauricio et al. (19). GP was measured using a pressure transducer and data logger (GN200, Sao Pualo, Brazil) in 120 mL serum bottles incubated at 39 °C for 24 h.

#### 2.5.1 Inoculum donor and preparation

The semi-automatic GP system equipped with a pressure transducer and data logger (Pressure Press Data GN200, São Paulo, Brazil), as described by Bueno et al. (20), was used to evaluate the dose–response effects of FDDP supplementation.

Ruminal content was freshly obtained from three slaughtered Holstein cows at the Faculty of Agriculture slaughterhouse, Alexandria University. Liquids and solids were placed in pre-warmed (39 °C) insulated flasks and immediately transported to the laboratory under anaerobic conditions. The pooled rumen contents consisted of equal volumes of rumen fluid and solid rumen material (50:50 v/v), which were squeezed through four layers of cheese cloth, maintained in a water bath at 39 °C, and saturated with CO_2_ until inoculation. In total, six analytical repetitions (six bottles/inoculum/treatment) were used; three for the determination of fermentation parameters and protozoal counts, and the remaining three for the determination of truly degraded organic matter (TDOM). Similarly, blank bottles (rumen fluid and buffer solution), as well as internal standard bottles (rumen inoculum, buffer solution, and clover hay), were prepared to correct for the sensitivity induced by inocula.

The samples (500 mg as-fed) of the incubated diets were accurately weighed into 120 mL glass bottles and incubated with 30 mL of buffer solution defined as MB9 (46). Then, 15 mL of the inoculum was added to the bottles, allowing a headspace of 75 mL. The buffer solution consisted of 2.8 g NaCl, 0.1 g CaCl_2_, 0.1 g MgSO_4_.7H_2_O, 2.0 g KH_2_PO_4_, and 6.0 g Na_2_HPO_4_ dissolved in distilled water and made up to a final volume of 1 L. After adjusting the pH to 6.8 using carbon dioxide flushing for 15 min, the bottles were sealed, manually mixed, and incubated at 39 °C for 24 h. Gas pressure in the headspace was measured at the intervals of 3, 6, 9, 12, and 24 h, and gas production was estimated using the following equation: *V* = 4.974 × *p* + 0.171 (*n* = 500; *r*^2^ = 0.98; data not reported), where *V* = gas volume (mL) and *p* = measured pressure (psi).

#### 2.5.2 Degradability and rumen fermentation profiles

At the end of the incubation period, all bottles were placed in cold water (4 °C) to stop the microbial fermentation process. After termination of the incubation at 24 h, truly degraded dry matter (TDDM) and TDOM were determined in three bottles for each inoculum by adding 50 mL of neutral detergent solution and incubating at 105 °C for 3 h. The bottle contents were filtered through pre-weighed crucibles and washed with hot water followed by acetone, and the residual DM and ash contents were determined thereafter. The partitioning factor (PF) was calculated as the ratio of mg of TDOM to gas volume (mL) at 24 h incubation (21). The contents of the remaining three bottles were used for determining fermentation characteristics. Rumen pH was measured directly within 2–3 min of sampling using a portable pH meter (GLP 21 model; CRISON, Barcelona, Spain). After incubation, the contents of each bottle were centrifuged at 3000 rpm for 15 min. The resulting supernatant was collected; a 5 mL aliquot was stored at −20 °C for subsequent ammonia analysis. Ruminal ammonia nitrogen (NH_3_-N) concentration was determined colorimetrically using a spectrophotometer, following the method described by Konitzer and Voigt (22). For short-chain fatty acid (SCFA) determination, the supernatant was prepared by acidification with metaphosphoric acid (4:1 ratio) and further centrifuged at 15,000 × g for 20 min at 4 °C before analysis using a gas chromatography (GC) system (Thermo Fisher Scientific, Inc., TRACE1300, Rodano, Milan, Italy) equipped with a capillary column and flame ionization detector. The analysis was carried out under a programmed temperature gradient with hydrogen as the carrier gas.

Methane (CH_4_) samples were collected from the incubation bottles at 3, 6, 9, 12, and 24 h using a 2 mL syringe and stored in evacuated Vacutainer tubes for analysis.

Methane concentration was determined using an Agilent 7,890 gas chromatograph equipped with a three-valve system, 1/8-inch packed columns with early back-flush of C6 components, and a thermal conductivity detector. Gas separation was achieved using a micro-packed column, with helium as the carrier gas at a flow rate of 28.0 mL/min. The detector and column temperatures were maintained at 250 °C and 60 °C, respectively. Linearity and calibration were established using a standard gas calibration curve covering the expected concentration range of the samples. Methane production at the end of incubation was calculated according to the method described by Tavendale et al. (23): CH_4_, mL = (total gas volume + headspace) × CH_4_ concentration, ml/ml. Both GP and CH_4_ were expressed as mL/g DM and mL/g TDOM after correcting the values of the total GP and incubated or truly degraded organic matter values for the corresponding blank.

In total, 2 mL of the rumen fluid subsample was mixed with 2 mL of methyl green-formalin-saline (MFS) solution and stored in a glass bottle at room temperature for subsequent total protozoal counting. Total protozoal counts were determined microscopically using an Olympus biological microscope (model CX31RBSFA, the Philippines) and a Neubauer improved bright-line counting chamber according to the procedure described by Dehority et al. (24).

### 2.6 Laboratory analyses

The FDDP product and experimental diets were oven-dried in a forced-air oven at 55 °C for 72 h, milled using a 2-mm screen, and analyzed according to the AOAC (25) procedures for dry matter (DM; method number 981.10), crude protein (CP; method number 967.03), ether extract (E. E; method number 963.15), and ash (method number 942.05). Fiber fractions, including NDF, acid detergent fiber (ADF), and acid detergent lignin (ADL), were determined using an ANKOM 200 Fiber Analyzer (ANKOM Technology Corporation, Macedon, NY, USA), following the method described by Van Soest et al. (26), and expressed exclusive of residual ash. Hemicellulose was calculated using the formula NDF minus ADF, while cellulose was derived by subtracting ADL from ADF.

The gross energy (GE) content of the FDDP and experimental diets was determined using an adiabatic oxygen bomb calorimeter (IKA® C 5000 Control, IKA-Werke GmbH & Co. KG, Staufen, Germany). The calorimeter was calibrated using benzoic acid as a standard reference material according to the manufacturer’s instructions (IKA-Werke GmbH & Co. KG, Staufen, Germany). Approximately 0.5–1.0 g of each dried and finely ground sample was weighed and completely combusted under excess oxygen pressure in a sealed combustion vessel. The values were expressed as MJ/kg dry matter (DM). Gross energy determination was conducted following standard procedures for feed evaluation as described by AOAC International (25).

### 2.7 Structure of the methanogenic community

#### 2.7.1 DNA extraction

Genomic DNA was extracted from the rumen samples using a CTAB-based method combined with phenol–chloroform purification. The extracted DNA was precipitated with ethanol, washed, and resuspended in DEPC water. DNA concentration was then determined using a NanoDrop spectrophotometer (Bio-Rad, USA) before storage at −20 °C.

#### 2.7.2 PCR amplification of purified rumen DNA

Methanogen-specific 16S rDNA fragments were amplified by nested PCR on the Agilent thermocycler 8,800 (Applied Biosystems, USA) using methanogen-specific forward and reverse primers. The first-round PCR was performed using the universal primers Met 86f [5′-GCTCAGTAACACGTGG-3′; (27)] and Met 915r [5′-GTGCTCCCCCGCCAATTCCT-3′; (28)] following the conditions described by Zhou et al. (47) under standard cycling conditions. The amplified products were separated and visualized on a 1.5% agarose gel and a UV transilluminator system (Gel Doc. Alphachem. Imager, USA), respectively, as shown in Figures 1A–C. The second-round PCR was then performed using the primers uniMet1-F (5′- CCGGAGATGGAACCTGAGAC-3′) and uniMet1-R (5′- CGGTCTTGCCCAGCTCTTATTC-3′) to increase specificity for methanogens, and the final products were confirmed by agarose gel electrophoresis.

**Figure 1:**
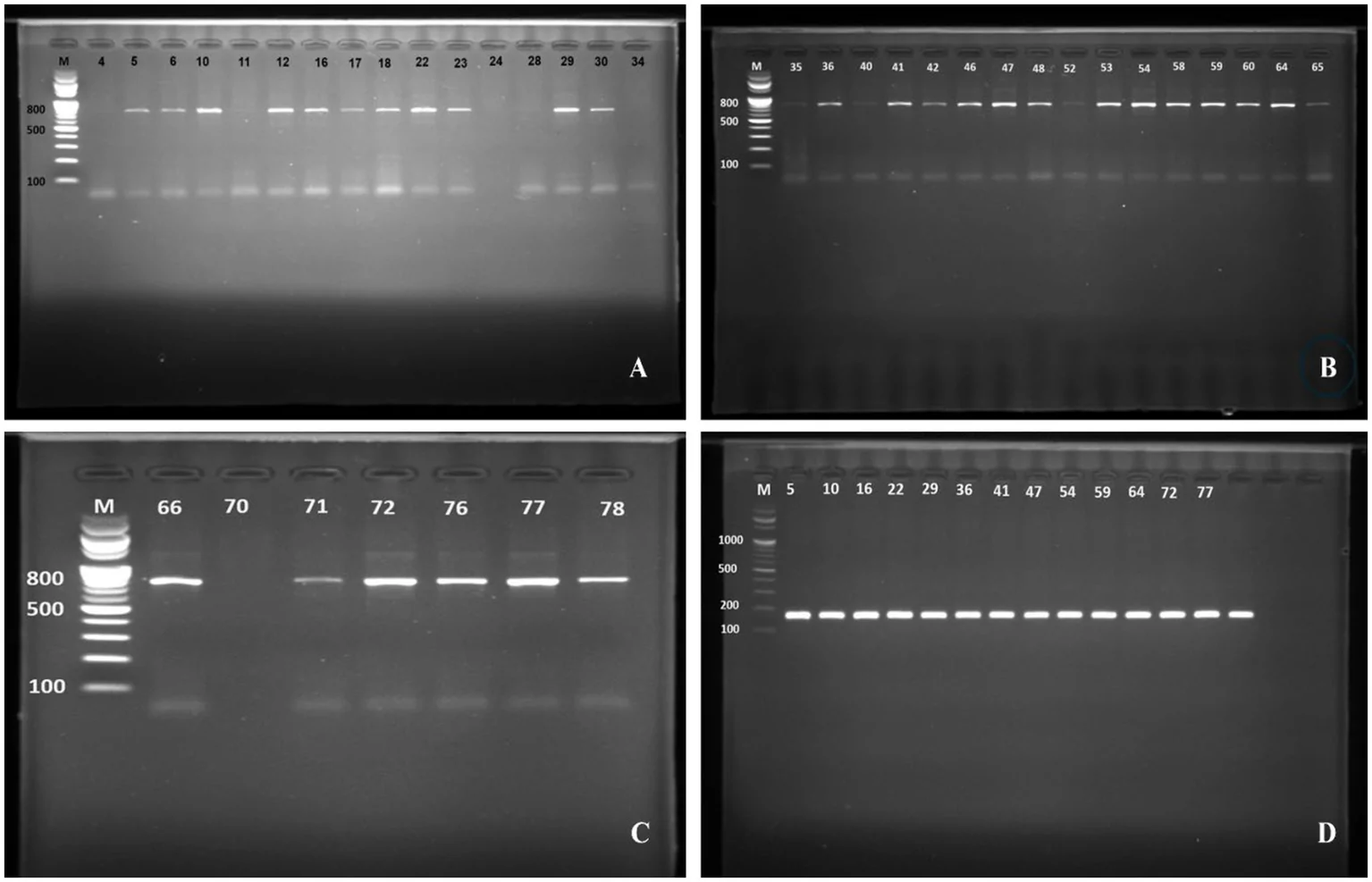
**(A–C)** PCR amplification of the rumen samples using long PCR primers, amplicon ~800 bp. Lanes M: 1 kbp DNA molecular marker. Lanes 1–78: The number of the rumen samples. **(D)** PCR amplification of the rumen samples using nested PCR primers, amplicon ~178 bp. Lanes M: 1 kbp DNA molecular marker. Lanes 5–77: The number of the randomly selected long PCR templates.

#### 2.7.3 DNA sequencing of nested PCR products

PCR products of approximately 179 bp were purified using the Qiagen PCR Purification Kit and subsequently submitted to Macrogen (Korea) for sequencing.

#### 2.7.4 DNA BLAST analysis and phylogenetic tree construction based on DNA sequences

Nucleotide sequences were analyzed using BLAST against the GenBank database (https://blast.ncbi.nlm.nih.gov/Blast.cgi), followed by multiple sequence alignment using Clustal W (https://www.genome.jp/tools-bin/clustalw). Phylogenetic relationships were then inferred using the MEGA11 software (https://www.megasoftware.net/).

### 2.8 Statistical analysis

Data from the *in vitro* assay were analyzed using the generalized linear model procedure of SAS (29). The following model was assumed: Y_ij_ = *μ* + F_i_ + e_ij_. The model included the overall mean (μ), treatment effect (F_i_), and random error term (e_ij_). Differences among treatment means were evaluated using Duncan’s multiple range test (30). The *in vitro* gas production experiment was performed in one run for all treatments. The analytical replicates were averaged before statistical analysis, with each inoculum being the statistical replicate; thus, the statistical replicates of the treatments (*n* = 3) were the true replicates. Orthogonal contrast statements were designed to test the linear and quadratic responses of each dependent variable to the increasing concentrations of fermented date palm stones.

## 3 Results

### 3.1 . *In vitro* experiment

The effects of different levels of FDDPs on rumen fermentation characteristics and protozoal counts are presented in Table 2. Linear and quadratic reductions (*p* = 0.001) were observed in GP, TDDM, and TDOM with increasing levels of FDDP supplementation. However, FDDP supplementation did not affectCH_4_, PF, total protozoal counts, and NH_3_ concentration compared to the control group. On the other hand, FDDP supplementation increased pH (linearly, *p* = 0.012; quadratically, *p* = 0.032) with increasing levels of FDDPs.

**Table 2:** Effects of FDDP supplementation levels on ruminal gas production (GP), methane production, pH, truly degraded organic matter (TDOM), partitioning factor, ruminal protozoal counts, and ammonia nitrogen (NH_3_-N) concentration after 24 h of incubation.

Items	Treatments					SEM	*p*-value	*p*-value
	CTRL	FDDP_25%_	FDDP_50%_	FDDP_75%_	FDDP_100%_		*L*	*L*
GP (ml/ gDM)	177.24	172.73	164.15	164.02	153.09	1.500	0.001	0.001
CH_4_ (mL/gDOM)	19.84	19.34	18.92	19.60	19.72	0.324	0.997	0.235
Rumen pH	6.32	6.30	6.34	6.36	6.49	0.023	0.012	0.032
TDDM (g/kg)	760.10	747.68	724.78	704.07	695.00	6.419	0.001	0.001
TDOM (g/kg)	769.98	755.52	741.97	705.92	703.17	6.576	0.001	0.001
PF (mg TDOM/mL GP)	3.80	3.75	3.90	3.70	4.02	0.040	0.174	0.234
Total protozoa (*×*10^5^/mL)	5.55	4.90	5.65	5.29	5.33	0.228	0.980	0.965
*Entodinium spp*	4.90	4.05	5.10	4.63	4.92	0.223	0.707	0.915
*Diplodinium Spp*	0.67	0.80	0.40	0.50	0.17	0.114	0.108	0.054
*Epidinium Spp*	0.60	ND	0.20	ND	ND	0.080	0.032	0.089
*Eudiplodinium Spp*	0.40	0.80	0.20	1.0	0.17	0.101	0.717	0.432
*Isotricha Spp*	ND	0.80	0.60	0.67	0.50	0.102	0.237	0.189
*Ophryscolex Spp*	0.40	0.27	0.40	0.17	0.33	0.085	0.703	0.861
NH_3_-N (mg/100 mL)	21.47	20.29	21.60	21.33	21.28	0.285	0.753	0.376

CTRL, 0% FDDP + 100% yellow corn, FDDP_25%_, 25% FDDP + 75% yellow corn, FDDP_50%_, 50% FDDP + 50% yellow corn, FDDP_75%_, 75% FDDP + 25% yellow corn, and FDDP_100%_:100% FDDP + 0% yellow corn. SEM, standard error of the mean; L, Linear; Q, quadratic.

The effects of FDDP on the ruminal molar proportions of individual SCFAs are shown in Table 3. No significant (*p* > 0.05) differences were observed in the molar proportions of the main SCFAs with FDDP supplementation. However, heptanoic molar proportions were linearly enhanced (*p* < 0.05) and tended to increase quadratically (*p* = 0.076) compared to the control group.

**Table 3:** Effects of FDDP supplementation on the ruminal molar proportions of individual SCFAs after 24 h of incubation.

Items, %	Treatments					SEM	*p*-value	
	CTRL	FDDP_25%_	FDDP_50%_	FDDP_75%_	FDDP_100%_		*L*	*Q*
Acetic	43.18	43.17	43.82	42.98	42.92	0.390	0.833	0.475
Propionic	24.88	24.67	24.26	24.83	24.91	0.668	0.979	0.813
Iso-butyric	2.14	2.11	2.21	2.32	2.31	0.071	0.293	0.131
n-butyric	19.45	20.06	19.60	19.80	19.94	0.221	0.650	0.465
Iso-valeric	5.18	5.08	5.14	5.26	5.45	0.128	0.453	0.378
n-valeric	4.11	3.83	3.92	3.79	3.64	0.109	0.224	0.232
Iso-caproic	0.02	0.08	0.05	0.02	0.04	0.012	1.000	0.916
Caproic	0.99	0.99	0.96	0.95	0.80	0.067	0.415	0.183
Heptanoic	0.14	0.13	0.14	0.15	0.13	0.013	0.029	0.076

CTRL, 0% FDDP + 100% yellow corn, FDDP_25%_, 25% FDDP + 75% yellow corn, FDDP_50%_, 50% FDDP + 50% yellow corn, FDDP_75%_, 75% FDDP + 25% yellow corn, and FDDP_100%_:100% FDDP + 0% yellow corn. SEM, standard error of the mean; L, Linear; Q, quadratic.

### 3.2 Structure of the methanogenic community

#### 3.2.1 PCR amplification of the rumen samples

PCR amplification of the rumen samples using long and nested PCR primers is shown in Figure 1.

#### 3.2.2 DNA sequencing and sequence analysis

The obtained DNA sequences were subjected to further analysis using DNABLAST on the NCBI, and the examined bacteria were identified based on the percentage of similarity with reference strains listed in the GenBank database, as shown in Figure 2.

**Figure 2:**
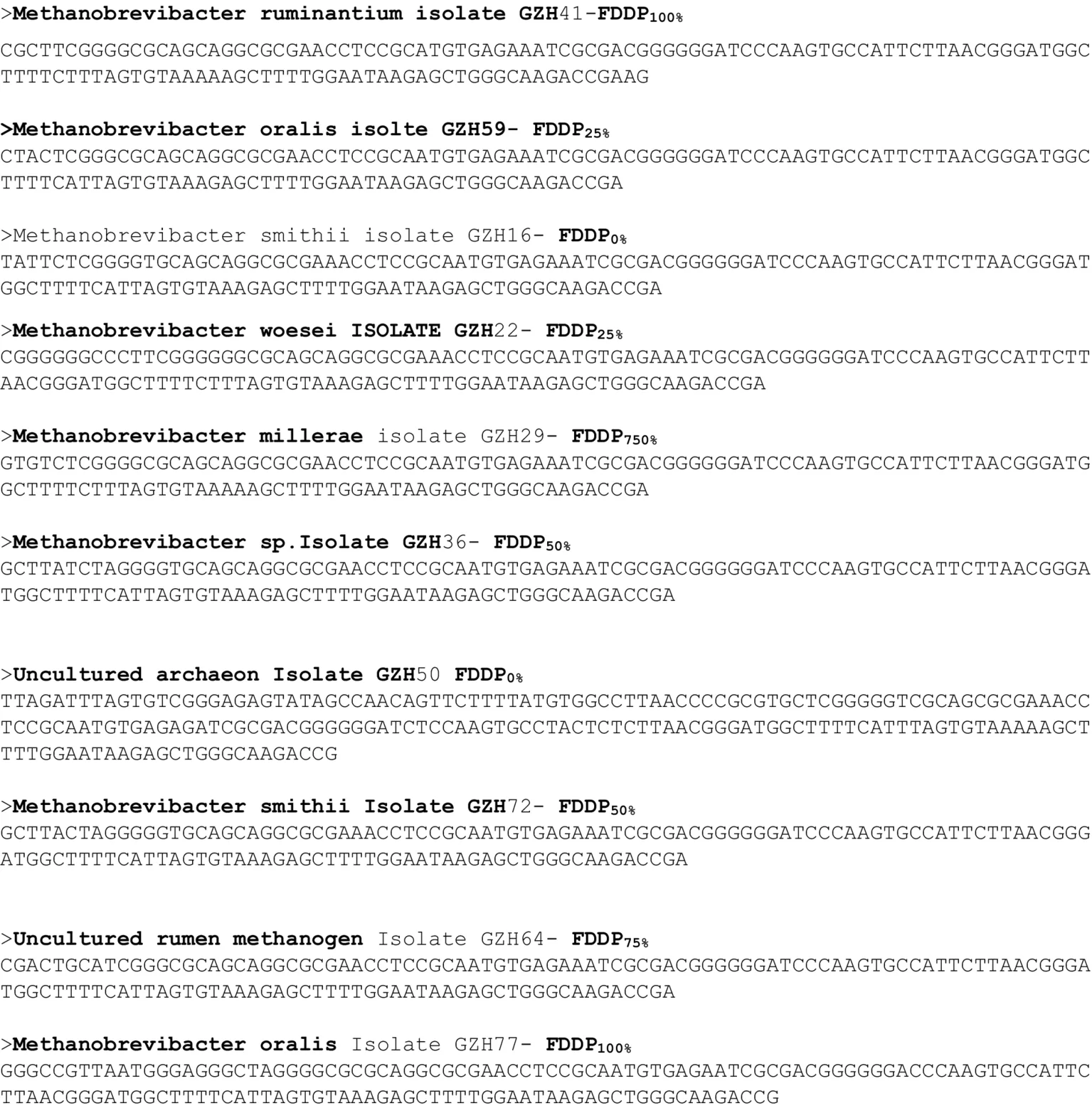
Obtained nucleotide sequences from selected rumen samples and their tentative identification based on BLAST analysis.

#### 3.2.3 Phylogenetic analysis

The phylogenetic relationships inferred from the neighbor-joining analysis based on the partial sequences of the 16S rRNA gene are depicted in Figure 3. The study revealed the presence of two distinct clusters. The largest cluster comprised 11 operational taxonomic units (OTUs) associated with *Methanobrevibacter*, forming two distinct subclusters with a bootstrap support value of 29%. The first subcluster within *Methanobrevibacter* included nine OTUs with nearly identical sequences, showing bootstrap support values ranging from 39 to 67%. The OTUs within this cluster were associated with different levels of date palm incorporation, including FDDP 0%, FDDP 25%, FDDP 75%, and FDDP 100%. However, the second subcluster diverged into two OTUs with a bootstrap support value of 58%, and the FDDP level represented in this cluster was 50%. The outgroup for the second cluster was the *Methanobrevibacter oralis* isolate, which was associated with the 100% FDDP treatment. These findings indicate the phylogenetic affiliation and variation among the detected methanogenic archaeal sequences rather than providing a comprehensive assessment of the overall methanogenic community structure.

**Figure 3:**
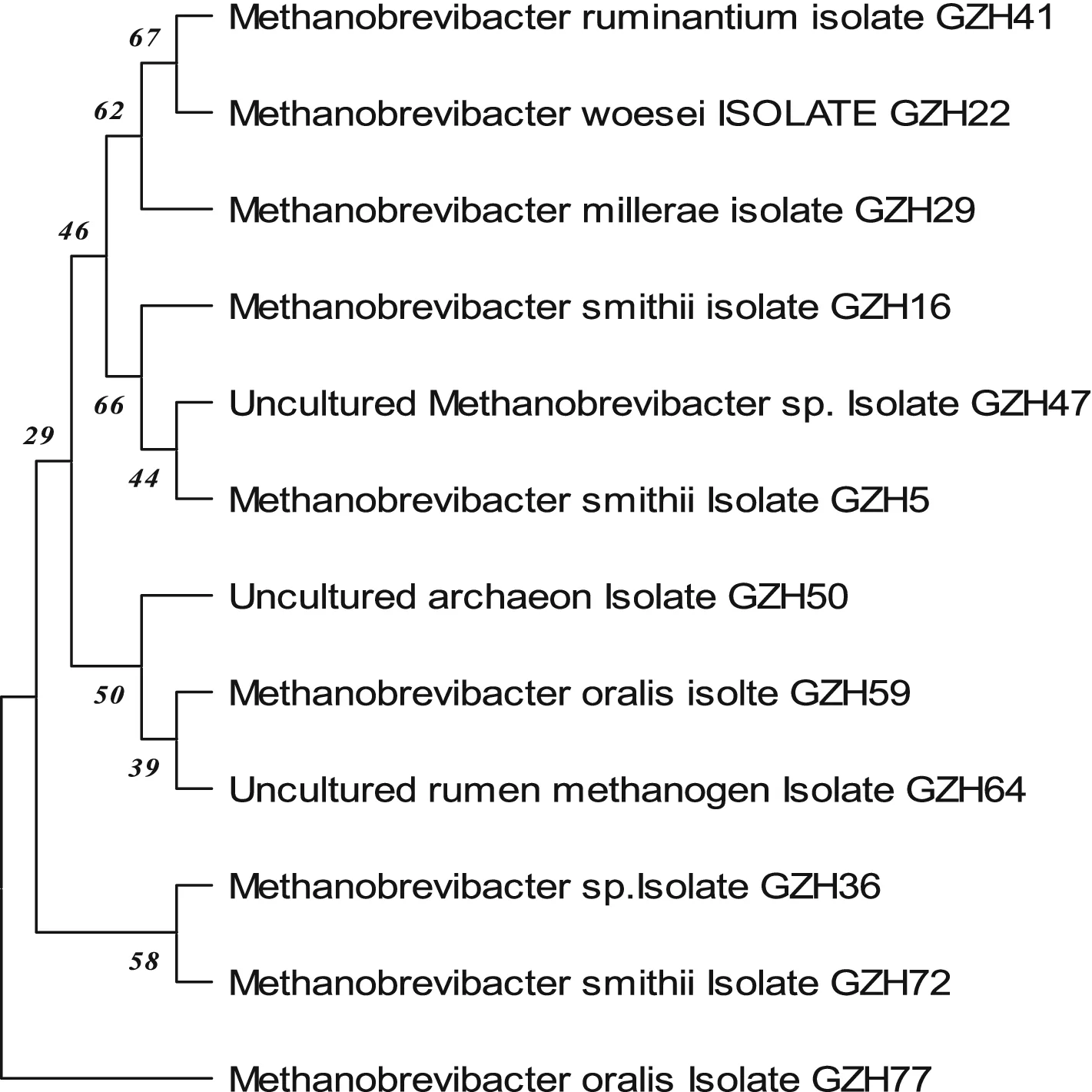
Phylogenetic tree of all sequenced rumen samples (16S rRNA gene partial sequences) using MEGA11 (the neighbor-joining tree method).

## 4 Discussion

Agricultural by-products can serve as viable feed alternatives in arid regions (31, 32), but their impact on animal performance and the rumen ecosystem must be carefully evaluated (3). Moreover, FDDP by-products, in particular, are rich in soluble carbohydrates, making them a valuable energy source, although their protein content is low.

The results of our experiment revealed that the replacement of corn grain with FDDP levels decreased *in vitro* ruminal GP compared to the control group. Our finding is consistent with that of Boufennara et al. (33), who reported that the highest cumulative gas production after 144 h of incubation was observed for Kentichi variety waste dates, while the lowest gas production was obtained with date pits.

In addition, the substitution of corn grain with FDDP levels resulted in linear and quadratic increases in ruminal pH. Our findings align with those of Martel et al. (34), who observed that higher dietary sugar concentrations increased ruminal pH in dairy cows. This effect may be due to sugar providing less fermentable carbon for fermentation acid production compared to starch (35). Moreover, rumen microbes can ferment sugars directly into microbial glycogen, serving as an intracellular energy reserve and further limiting the formation of fermentation acids (36). In addition, the elevated levels of ADF and NDF in date pit-based diets contributed to maintaining a more neutral rumen pH (37).

In this study, increasing the replacement of corn grain with FDDP resulted in reduced *in vitro* TDDM and TDOM. Similar findings were reported by Alhomidy et al. (48), who evaluated discarded dates as an alternative feed source in Najdi lamb diets, replacing 0%, 15%, and 30% of the concentrate feed. These effects may be related to the high lignin and fiber content of date pits, which can restrict microbial degradation and fermentation efficiency, thereby contributing to the low gas production observed (33).

In addition, higher levels of fiber components such as NDF, ADF, and lignin were associated with lower gas production, likely because highly lignified materials are less accessible to rumen microbes. Furthermore, date by-products may contain phenolic compounds and condensed tannins, which can also reduce ruminal degradation efficiency (38). The previous interpretation is consistent with the results obtained in this study, as total gas production decreased linearly with increasing FDDP substitution rates.

Replacing corn grain with FDDP had little effect on the concentrations of most short-chain fatty acids, except for heptanoic acid, which increased with increasing levels of FDDP inclusion. Similar observations have been reported in previous studies (39, 40). These results indicate a shift in ruminal fermentation pathways toward greater butyrate production in diets rich in soluble carbohydrates. Furthermore, the results may indicate alterations in ruminal fermentation pathways to accommodate the increased hydrogen flux generated from rapidly fermentable sugar sources or enhanced conversion of lactate into butyrate in sheep receiving soluble carbohydrate-rich diets, as proposed by Khattab and Anele (41).

In our study and based on the sequence similarity of methanogenes, the majority of the sequences were linked to *Methanobrevibacter* spp. Despite the similarity percentages between the various sequences being modest, ranging from 39 to 67%, there were no notable differences in methane production when using varying levels of FDDP. The replacement ratio of FDDP did not affect methanogen diversity, which is consistent with a previous study (12). Our results are consistent with those of Rabee et al. (42), who observed no clear relationship between methanogen abundance and methane emissions in the rumen.

## 5 Conclusion

Under the experimental conditions, the results suggest that FDDP is a promising alternative energy source that can partially or totally replace yellow corn grain without detrimental effects on rumen fermentation characteristics and the methanogen community in the feeding of ruminants. In addition, substituting corn with FDDP as an agricultural waste material may help reduce concentrate feed costs, thereby offering potential economic benefits. Further *in vivo* research is necessary to validate these findings and to better assess the practical application of FDDP in ruminant nutrition.

## Data Availability

The data analyzed in this study are available in the NCBI Genbank under accession number MK138351: https://www.ncbi.nlm.nih.gov/nuccore/MK138351.
